# Comparative Analysis of Volatile Organic Compounds in Herbaceous and Fruity Pitaya (*Selenicereus undatus*) Cultivars During Ripening via GC × GC-TOF-MS

**DOI:** 10.3390/foods15213917

**Published:** 2026-11-02

**Authors:** Zhi-Jun Yang, Jie-Dong Chen, Jun-Cheng Li, Si-Yu Huang, Meng-Tao Yang, Hong-Hua Su, Qing-Ming Sun

**Affiliations:** 1 Institute of Fruit Tree Research, Guangdong Academy of Agricultural Sciences, Key Laboratory of South Subtropical Fruit Biology and Genetic Resource Utilization, Ministry of Agriculture and Rural Affairs, Guangdong Provincial Key Laboratory of Science and Technology Research on Fruit Tree, Guangzhou 510640, China; yangzhijun@gdaas.cn (Z.-J.Y.); lijuncheng@gdaas.cn (J.-C.L.); 2 Department of Nutrition, School of Public Health, Sun Yat-Sen University, Guangzhou 510080, China; chenjd36@mail2.sysu.edu.cn (J.-D.C.); huangsy9@mail2.sysu.edu.cn (S.-Y.H.); yangmt27@mail.sysu.edu.cn (M.-T.Y.); 3 College of Plant Protection, Yangzhou University, Yangzhou 225009, China; 4 Proteomics and Metabolomics Facility, Institute of Biotechnology, Cornell University, Ithaca, NY 14850, USA

**Keywords:** pitaya, herbaceous aroma, fruity aroma, GC × GC-TOF-MS, ROAVs

## Abstract

Different pitaya cultivars (*Selenicereus undatus*) have unique aroma characteristics. For example, the “Vietnamese White Heart” pitaya fruit exhibits a distinct “herbaceous aroma”, while the new variety “Yuehong No. 6” exhibits a “fruity aroma”, but the underlying mechanisms remain unclear. This study aimed to characterize and compare the developmental changes in volatile organic compound (VOC) profiles between two pitaya cultivars at 25, 30, and 35 days after flowering (DAF), using GC × GC-TOF-MS combined with relative odor activity value (ROAV) analysis. The results show that the ROAVs of 2-pentylfuran and (E)-2-octenal increased progressively in “Vietnamese White Heart” but decreased during development in “Yuehong No. 6”. Although their ROAVs were higher in “Yuehong No. 6” at 25 and 30 DAF, this pattern was reversed at 35 DAF. At 35 DAF, 2-undecanone also showed a higher ROAV in “Vietnamese White Heart”, whereas 2-dodecenal showed a higher ROAV in “Yuehong No. 6”. This study reveals cultivar-dependent developmental trajectories of several potentially aroma-relevant VOCs, and provides a basis for future flavor-oriented molecular breeding, harvest timing optimization and fruit processing.

## 1. Introduction

Pitaya (*Selenicereus undatus*), a fruit native to the tropical and subtropical regions from Central America to northern South America, is now widely cultivated and distributed in China, particularly in provinces such as Guangdong, Guangxi, Hainan, and Fujian [1,2,3,4]. It is rich in polyphenols, flavonoids, terpenes, minerals, betalains, vitamins, fatty acids and sterols [5,6]. These bioactive compounds confer a range of health benefits, such as antioxidant, anti-inflammatory, antifungal, antiulcer, antidiabetic, antimicrobial, anticancer, diuretic, hypolipidemic, hepatoprotective and neuroprotective effects [7,8]. Additionally, pitaya has been incorporated into cosmetic formulations, such as natural skin-whitening agents and natural lipsticks [9,10]. Owing to its exceptional nutritional value and commercial potential, increasing attention has focused on its composition and sensory attributes. For example, our previous study investigated the potential mechanisms underlying quality deterioration during pitaya storage [11]. Earlier research elucidated the mechanism of color formation in both the flesh and peel of pitaya [12]. In addition, other studies have also explored changes in metabolites during pitaya development and ripening [13]. However, research on the complex aroma characteristics of pitaya fruit and its volatile organic compounds (VOCs) remains very limited.

Flavor plays a critical role in fruit quality evaluation and encompasses aroma, taste and other sensory attributes [14]. Among these, aroma is directly perceived by consumers and is a key factor affecting consumer acceptance and market competitiveness [15]. Fruit aroma results from a complex mixture of VOCs, mainly including alcohols, olefins, aldehydes, esters, phenols, terpenes and ketones [16,17]. Although VOCs constitute only 0.001–0.01% of the fresh fruit’s weight, they have a significant impact on its aroma quality [18]. Notably, differences in fruit aroma are closely related to the composition and abundances of VOCs, which vary with species and varieties [16]. A variety of aroma-related substances have been identified in many fruits, such as apples, strawberries, grapes and bananas [19,20,21,22]. During pitaya cultivation, our team discovered that the “Vietnamese White Heart” pitaya fruit has a typical, slightly unripe “herbaceous aroma”, while our new pitaya variety, “Yuehong No. 6”, has a pleasant, sweet “fruity aroma”. Although consumers and breeding experts can easily perceive these differences through sensory perception, the specific key VOCs that contribute to this differentiation between herbaceous and fruity aromas have not yet been precisely identified.

To address the shortcomings in understanding these phenotypic differences, this study aimed to comprehensively analyze the flavor dynamics of these two different varieties during key maturity stages (25, 30, and 35 days after flowering (DAF)). This study used two-dimensional gas chromatography with time-of-flight mass spectrometric detection (GC × GC-TOF-MS). This technique overcomes the resolution limitations of traditional one-dimensional GC-MS, significantly improving the peak capacity and signal-to-noise ratio of trace aroma compounds, and enabling precise analysis of complex volatile components. Combining high-resolution multidimensional data with ROAV assessment aimed to accurately identify the evolutionary trajectories and potential volatile markers associated with the differences between herbaceous and fruity aromas. This research not only provides a scientific basis for a deeper understanding of pitaya fruit flavor compounds and secondary metabolic networks (such as the lipoxygenase pathway), but also offers a theoretical foundation for future targeted breeding to improve the sensory quality of pitaya fruit, the development of optimal harvesting standards, and the industrial processing of pitaya fruit flavor derivatives.

## 2. Materials and Methods

### 2.1. Sampling and Sensory Evaluation

**Figure 1:**
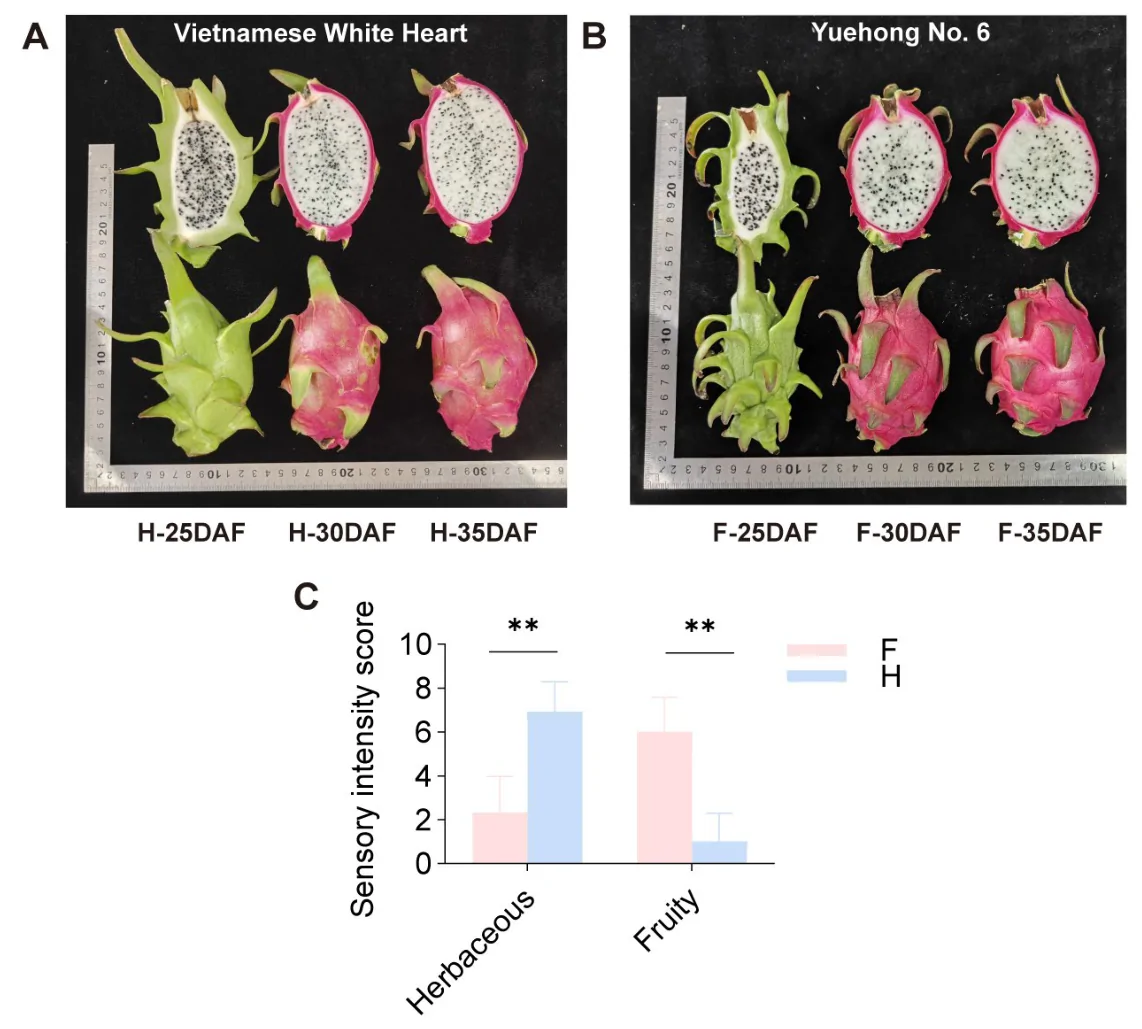
Fruit appearance and sensory characteristics of two pitaya cultivars. (**A**) Representative fruits of “Vietnamese White Heart” (Group H). (**B**) Representative fruits of “Yuehong No. 6” (Group F). (**C**) Herbaceous- and fruity-aroma intensity scores of fruits harvested at 35 DAF. Data are presented as mean ± SD (*n* = 10 panelists). ** *p* < 0.01, as determined using a two-tailed paired Student’s *t*-test.

Two cultivars of pitaya with different aromas were collected from the germplasm nursery of the Institute of Fruit Tree Research, Guangdong Academy of Agricultural Sciences. During routine breeding and cultivar evaluation, “Vietnamese White Heart” and “Yuehong No. 6” were consistently described by the research team as exhibiting predominantly herbaceous and fruity aroma characteristics, respectively. These terms are used here as operational cultivar descriptors. The “Vietnamese White Heart” cultivar exhibits an “herbaceous” aroma upon ripening (Group H), while the “Yuehong No. 6” cultivar exhibits a “fruity” aroma (Group F). Pitaya fruits from both groups were harvested at three developmental stages: 25, 30, and 35 days after flowering (DAF). During ripening, the peel color of both pitaya fruits with different aroma characteristics changes from green to purple-red. The fruit with a fruity aroma has a significantly darker color and is relatively smaller. The flesh of both fruits is white (Figure 1A,B). For each stage, fifteen normally developed fruits were randomly selected. The flesh of each fruit was halved longitudinally, and one half was cut again along the same axis. One-quarter of the flesh (1/4 of the whole fruit) was taken as a sample. Flesh portions from five fruits were pooled to form a composite sample, and three biological replicates were prepared per group.

Sensory evaluation was conducted using fruit harvested at 35 DAF, corresponding to the fully ripe stage. Fruits at 25 and 30 DAF had not yet reached full ripeness and were therefore excluded from sensory evaluation to ensure that the assessment reflected the characteristic aroma profiles of fully ripe fruits. Ten panelists aged 18–60 years were recruited based on the following criteria: good general health, normal gustatory and olfactory function, and no history of taste or olfactory disorders. Prior to evaluation, all panelists received consistent instructions on the definitions of herbaceous and fruity aroma attributes and the use of the 0–9 intensity scale, where 0 indicated that the attribute was not perceived and 9 indicated extremely high intensity. Sensory assessments were conducted at room temperature. Samples of “Vietnamese White Heart” (Group H) and “Yuehong No. 6” (Group F) were prepared in triplicate, assigned blinded identification codes, and presented to panelists in randomized order. Each panelist independently evaluated the intensity of herbaceous and fruity aroma attributes by tasting the fruit pulp.

### 2.2. Chemicals

N-hexyl-d13 alcohol was purchased from C/D/N Isotopes Inc. (Pointe-Claire, QC, Canada). Saturated Alkanes was obtained from Sigma-Aldrich Company (St. Louis, MO, USA). N-hexane was acquired from Yonghua Chemical Co., Ltd. (Shanghai, China).

### 2.3. GC × GC-TOF-MS Analysis

#### 2.3.1. Extraction of VOCs

An appropriate amount of pitaya pulp was transferred into a 15 mL centrifuge tube and frozen in liquid nitrogen for 5 min. The frozen material was homogenized for 3 min, after which 1.00 g of the homogenized sample was transferred into a 20 mL headspace vial. A total of 10 μL of n-hexyl-d13 alcohol internal-standard solution (1 mg/L in 50% aqueous ethanol) was added to each vial.

A 50/30 μm divinylbenzene/carboxen/polydimethylsiloxane (DVB/CAR/PDMS) solid-phase microextraction (SPME) fiber (1 cm fiber length, model 57329 U; Supelco, Bellefonte, PA, USA) was used for VOC extraction. This triple-phase coating was selected for its broad extraction coverage of volatile compounds with different polarities and volatilities. Before extraction, the SPME fiber was conditioned in a heating chamber at 270 °C for 10 min. VOC extraction was performed at 60 °C for 30 min to facilitate the release of volatile compounds from the pitaya pulp matrix and ensure sufficient enrichment for GC × GC-TOF-MS analysis. Subsequently, the fiber was inserted into the GC injector port and desorbed the analytes at 250 °C for 5 min, followed by reconditioning at 270 °C for 10 min. Finally, 10 μL of a saturated alkane solution was pipetted into a separate 20 mL headspace vial, and incubation, extraction, and injection were performed using identical conditions.

#### 2.3.2. Detection Conditions

VOCs were analyzed using a LECO Pegasus^®^ BT 4D system (LECO, St. Joseph, MI, USA) integrated with an Agilent 8890A gas chromatograph (Agilent Technologies, Palo Alto, CA, USA). The system featured a split/splitless injector, a dual-stage cryogenic modulator (LECO), and a time-of-flight mass spectrometer (TOF-MS). Separation was achieved using a two-dimensional column setup: a primary column (1D) Rxi-5Sil MS column (30 m × 250 μm i.d., 0.25 μm film thickness; Restek, Bellefonte, PA, USA), and a secondary column (2D) Rxi-17Sil MS column (2.0 m × 150 μm i.d., 0.15 μm film thickness; Restek, Bellefonte, PA, USA). High-purity helium gas (>99.999%) served as the carrier, maintained at a constant flow rate of 1.0 mL/min.

The gas chromatograph (GC) oven was programmed as follows: initial temperature of 40 °C held for 3 min, ramped at 4 °C/min to 200 °C, followed by a further increase at 10 °C/min to 250 °C, which was held for 5 min. The secondary oven temperature was maintained 5 °C higher than the primary oven, and the modulator temperature was set 15 °C above the secondary oven. The modulation period was 6 s. The GC injector temperature was maintained at 250 °C.

Mass spectrometric detection was performed in electron ionization (EI) mode at 70 eV, with a detector voltage of 2016 V and a scan range of m/z 35–550. Both the transfer line and ion source temperatures were set to 250 °C. Data acquisition was performed at a rate of 200 spectra per second.

VOC abundances were semi-quantitatively estimated using n-hexyl-d13 alcohol as a single internal standard. Given the differences in detector response factors and SPME extraction efficiencies among compounds, the resulting values were expressed as relative abundances rather than absolute concentrations.

#### 2.3.3. ROAV Calculation and Screening of Potential Aroma-Relevant VOCs

The relative odor activity value (ROAV) was calculated to estimate the relative contribution of individual VOCs to the overall volatile profile. According to the previously reported method [23], the compound considered to make the greatest contribution to the overall aroma of each sample was defined as the reference compound and assigned an ROAV of 100 (ROAV*_Max_* = 100). The ROAVs of the remaining VOCs were calculated as follows:

$${ROAV}_{D}=100\;\times \frac{C_{D}}{C_{Max}}\times \frac{T_{Max}}{T_{D}}$$

where *C_D_* and *T_D_* represent the relative content and odor threshold of compound D, respectively, whereas *C_Max_* and *T_Max_* represent the relative content and odor threshold of the reference compound assigned an ROAV of 100. Odor thresholds were obtained from published literature and/or established flavor databases. Compounds with ROAV ≥ 1 were regarded as potential major aroma contributors, whereas compounds with 0.1 ≤ ROAV < 1 were considered potential aroma modifiers. Accordingly, the calculated ROAVs should be interpreted as relative indicators of potential aroma contributions rather than absolute measures of sensory impact.

### 2.4. Data Processing and Statistical Analysis

Flavoromics data were analyzed using ChromaTOF software (version 5.0; LECO Corporation, St. Joseph, MI, USA), which provided detailed information including compound names, retention times, CAS registry numbers, database-derived retention indices (RIs), experimentally calculated RI values using the normal alkanes C7–C30, and corresponding peak areas. These datasets were integrated to generate the final analytical outputs. VOCs were tentatively annotated based on mass spectral information and retention indices calculated using C7–C30 n-alkanes. Relative abundances were estimated using chromatographic peak responses normalized to the internal standard. As no reference standards were used for comparison, and no compound-specific calibration curves were established, subsequent research should focus on the qualitative findings. Odor descriptors and sensory profiles were collected from published literature [24] and online resources (https://cosylab.iiitd.edu.in/flavordb/, accessed on 30 January 2024).

For statistical evaluation, principal component analysis (PCA) was first employed to examine clustering trends among pitaya samples. To further improve class discrimination and identify characteristic volatile compounds, partial least squares discriminant analysis (PLS-DA) was performed. PLS-DA models were evaluated using cross-validation and 200 permutation tests to assess model performance and statistical significance. Three-fold cross-validation was applied to the cultivar comparisons at individual developmental stages. Considering the relatively limited biological sample size, future studies with additional replicates may further strengthen model robustness. Differential VOCs were selected based on VIP scores (VIP > 1) derived from the PLS-DA model, combined with a significance threshold of *p* < 0.05. Heatmaps were subsequently constructed to visualize these differences.

All additional statistical analyses and graphical representations were carried out using GraphPad Prism 10.1.2 (GraphPad Software Inc., San Diego, CA, USA). Significance testing was performed using Student’s *t*-test and analysis of variance (ANOVA), with *p* < 0.05 considered statistically significant.

## 3. Results

### 3.1. Developmental Changes in VOCs in Herbaceous-Aroma Pitaya During Ripening

Sensory evaluation results indicate significant differences in aroma characteristics between the two pitaya cultivars (Figure 1C). Group H exhibited significantly higher herbaceous-aroma intensity, whereas Group F displayed significantly higher fruity-aroma intensity (*p* < 0.01). These results provide direct sensory evidence for the distinct herbaceous and fruity aroma profiles exhibited by the two cultivars.

#### 3.1.1. GC × GC-TOF-MS Analysis of VOCs in Herbaceous-Aroma Pitaya

**Figure 2:**
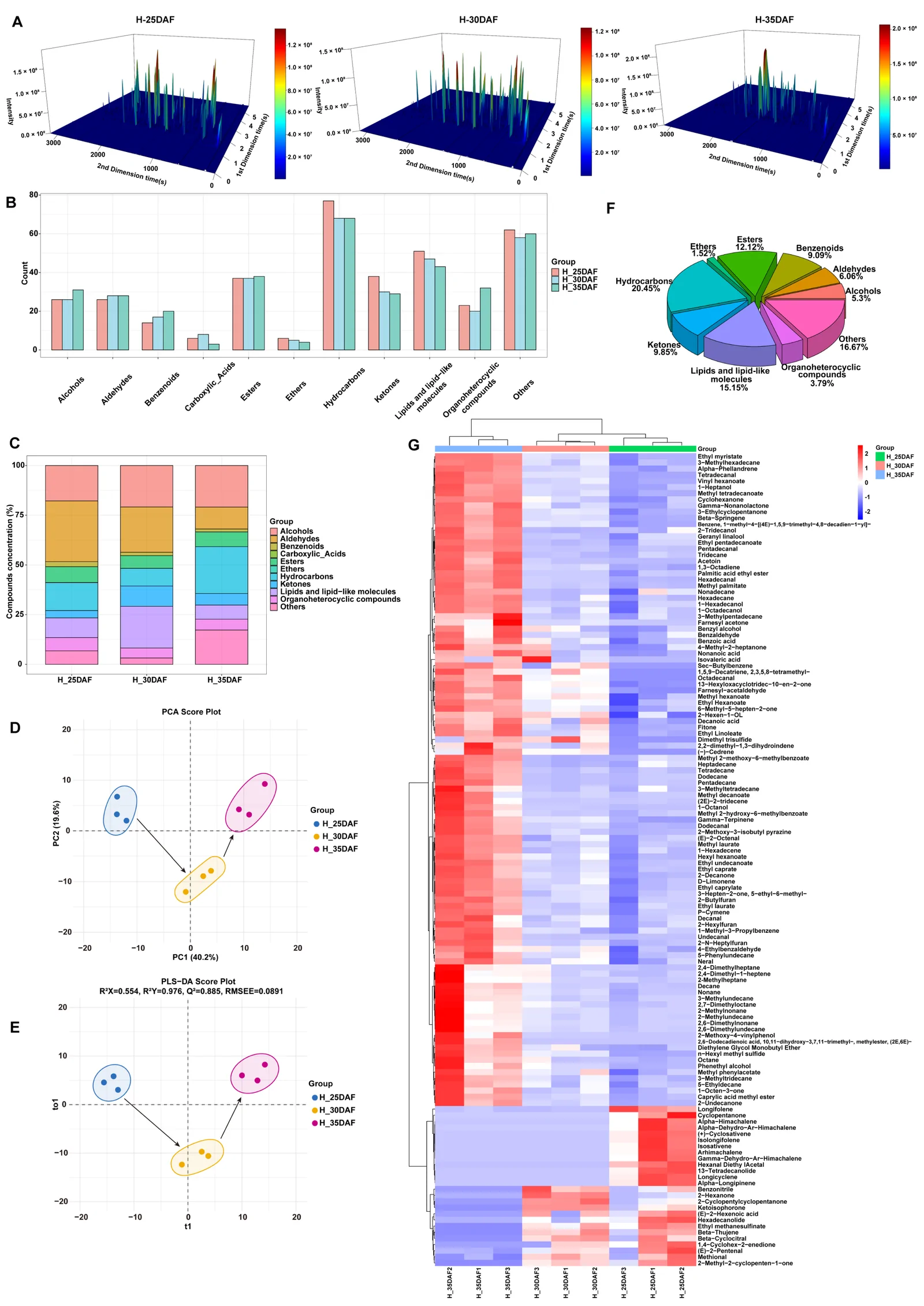
VOC analysis of herbaceous-aroma pitaya at 25, 30 and 35 DAF. (**A**) Three-dimensional chromatograms. (**B**) Number of VOCs in each class. (**C**) Relative abundance of each VOC class. (**D**) PCA score plot. (**E**) PLS-DA score plot. (**F**) The pie chart of the proportion of key differential VOC classes. (**G**) The cluster heatmap of key differential VOCs.

The changes in the types and quantities of VOCs are closely related to aroma characteristics [25]. VOCs in the herbaceous-aroma pitaya at 25, 30 and 35 DAF were analyzed using GC × GC-TOF-MS. The results are presented as three-dimensional chromatograms, with first-dimension retention time (s) on the horizontal axis and second-dimension retention time (s) on the vertical axis, effectively separating and classifying the VOCs (Figure 2A). To further illustrate the differences at different ripening stages of the herbaceous-aroma pitaya, the number of detected VOCs and their relative class proportions are shown in Figure 2B,C, respectively.

At 25 DAF, 366 VOCs were identified, comprising 26 alcohols, 26 aldehydes, 14 benzeneoids, 6 carboxylic acids, 37 esters, 6 ethers, 77 hydrocarbons, 38 ketones, 51 lipids and lipid-like molecules, 23 organoheterocyclic compounds, and 62 other compounds. At 30 DAF, 344 VOCs were detected, including 26 alcohols, 28 aldehydes, 17 benzeneoids, 8 carboxylic acids, 37 esters, 5 ethers, 68 hydrocarbons, 30 ketones, 47 lipids and lipid-like molecules, 20 organoheterocyclic compounds, and 58 other compounds. At 35 DAF, 356 VOCs were identified, consisting of 31 alcohols, 28 aldehydes, 20 benzeneoids, 3 carboxylic acids, 38 esters, 4 ethers, 68 hydrocarbons, 29 ketones, 43 lipids and lipid-like molecules, 32 organoheterocyclic compounds, and 60 other compounds (Figure 2B).

The relative composition of VOC classes varied across the three developmental stages. Alcohols were relatively high and increased over time. In contrast, aldehydes and benzeneoids decreased over time, with aldehydes showing the largest change. Esters, hydrocarbons and organoheterocyclic compounds initially decreased and then increased, with hydrocarbons showing the largest change. One study found that ester VOCs help enhance the fresh flavor of fruit, and there is a significant correlation between green and grassy aromas [26]. Ketones, lipids and lipid-like molecules initially increased and then decreased. Carboxylic acids and ethers remained consistently very low throughout ripening. These descriptive results indicate developmental variation in VOC-class composition (Figure 2C).

#### 3.1.2. Analysis of VOCs in Herbaceous-Aroma Pitaya

Principal component analysis (PCA) and partial least squares discriminant analysis (PLS-DA) were used to identify VOCs that significantly changed during the ripening of the herbaceous-aroma pitaya cultivar. The PCA score plot showed clear separation of VOCs at different ripening stages. The first two principal components accounted for approximately 59.8% of the total variation, with PC1 and PC2 explaining 40.2% and 19.6% (Figure 2D), respectively. These results indicate distinct differences in the volatile profiles of pitaya at 25, 30 and 35 DAF. The PLS-DA model was used to further evaluate the VOC differences across ripening stages. The PLS-DA score plot similarly revealed clear separation between stages, with R^2^X = 0.554, R^2^Y = 0.976, Q^2^ = 0.885, and RMSEE = 0.0891 (Figure 2E). Overall, both PCA and PLS-DA confirmed that VOC differences between 25, 30, and 35 DAF became more pronounced as ripening progressed.

Using VIP > 1 and *p* < 0.05 as screening criteria, a total of 132 differentially abundant VOCs were identified across 25, 30 and 35 DAF. These included 7 alcohols, 8 aldehydes, 12 benzeneoids, 16 esters, 2 ethers, 27 hydrocarbons, 13 ketones, 20 lipids and lipid-like molecules, 5 organoheterocyclic compounds, and 22 other compounds. Hydrocarbons (20.45%), lipids and lipid-like molecules (15.15%), and esters (12.12%) were the major differentially expressed components, followed by ketones (9.85%), benzenoids (9.09%), aldehydes (6.06%) and alcohols (5.3%) (Figure 2F).

Cluster heatmaps show significant changes in relative VOC abundances at different ripening stages (Figure 2G). As ripening progressed, the abundances of some key VOCs—particularly alcohols and esters—increased markedly. The levels of 1-heptanol, 1-hexadecanol, 1-octanol, 2-hexen-1-ol, 2-tridecanol, ethyl caprate, ethyl hexanoate, ethyl laurate, ethyl myristate, methyl 2-hydroxy-6-methylbenzoate, caprylic acid methyl ester, and palmitic acid ethyl ester gradually increased over time. In addition, the content of some hydrocarbons and ketones, such as (2E)-2-tridecene, 2,4-dimethylheptane, decane, dodecane, tetradecane,1-octen-3-one, 2-undecanone, and 6-methyl-5-hepten-2-one, also showed an increasing trend. In contrast, some VOCs decreased significantly during ripening, such as longicyclene, longifolene, (+)-cyclosativene, and (E)-2-pentenal. For example, the FDR-adjusted *p*-values for 1-heptanol, ethyl caprate, 2-undecanone, and longifolene were 0.00097, 0.00476, 0.00482, and 0.00178, respectively. Overall, these findings indicate marked compositional shifts in VOCs among the ripening stages of the herbaceous-aroma pitaya.

#### 3.1.3. ROAV Analysis in Herbaceous-Aroma Pitaya

Human perception of fruit aroma results from the interaction between G protein-coupled odorant receptors and VOCs released by the fruit. Fruit aroma is affected only when VOCs exceed a certain threshold concentration [27,28]. To assess the relative impact of each compound, the relative odor activity value (ROAV) is commonly applied. Compounds with higher ROAVs exert stronger effects on the overall volatile profile. In particular, VOCs with ROAVs ≥ 1 are regarded as the main contributors, while those with values around 100 are identified as the most dominant components. VOCs with an ROAV between 0.1 and 1 are classified as modifiers [27,29].

Table 1 presents the potential aroma-active compounds with ROAVs ≥ 0.1 in pitaya at different ripening stages. At 25, 30, and 35 DAF, 6, 11, and 12 compounds exhibited ROAVs above this threshold, respectively. Notably, 2-nonenal (ROAV = 100), characterized by a fatty and cucumber-like aroma, was the most prominent contributor throughout ripening. One study found that 2-nonenal is the second key flavor compound in both bayberry juice and bayberry fruit [30]. Additionally, 2-nonenal is also an important VOC in *Rosa roxburghii* fruit, suggesting that 2-nonenal is an essential pleasant property [31]. Moreover, 2-nonenal is an important component of natural fragrances [26]. The ROAVs of 2-pentylfuran and (E)-2-octenal increased markedly over time, reaching 19.83 and 10.83, respectively, at 35 DAF, indicating their potential role as key contributors to the grassy aroma. A previous study found that 2-pentylfuran, which has a green aroma, could be used to distinguish between yellow- and green-fleshed kiwifruit varieties [32]. Another study revealed that the content of 2-pentylfuran increased in deteriorated Chinese bayberry fruits, which may be one of the reasons for the off-flavor [33]. In addition, 2-undecanone, 1-hexanol, 2-ethylhexanol, and (2E,6Z)-nona-2,6-dienal may also contribute to grassy aroma. The ROAVs of heptanal exhibited an initial increase followed by a decline, with citrus, fatty, and rancid aroma characteristics. Furthermore, 1-octen-3-one has a mushroom-like aroma. It is a novel natural product used as a flavoring agent in the food industry [34]. In our study, the high ROAV of 1-octen-3-one and 2-heptanone may be related to the mushroom-like odor. 1-Octen-3-one was also detected in the yellow-fleshed peach “Jinyuan” and the white-fleshed peach “Hujing Milu” [34], which may contribute to a more complex overall volatile profile.

### 3.2. Developmental Changes in VOCs in Fruity-Aroma Pitaya During Ripening

#### 3.2.1. GC × GC-TOF-MS Analysis of VOCs in Fruity-Aroma Pitaya

**Figure 3:**
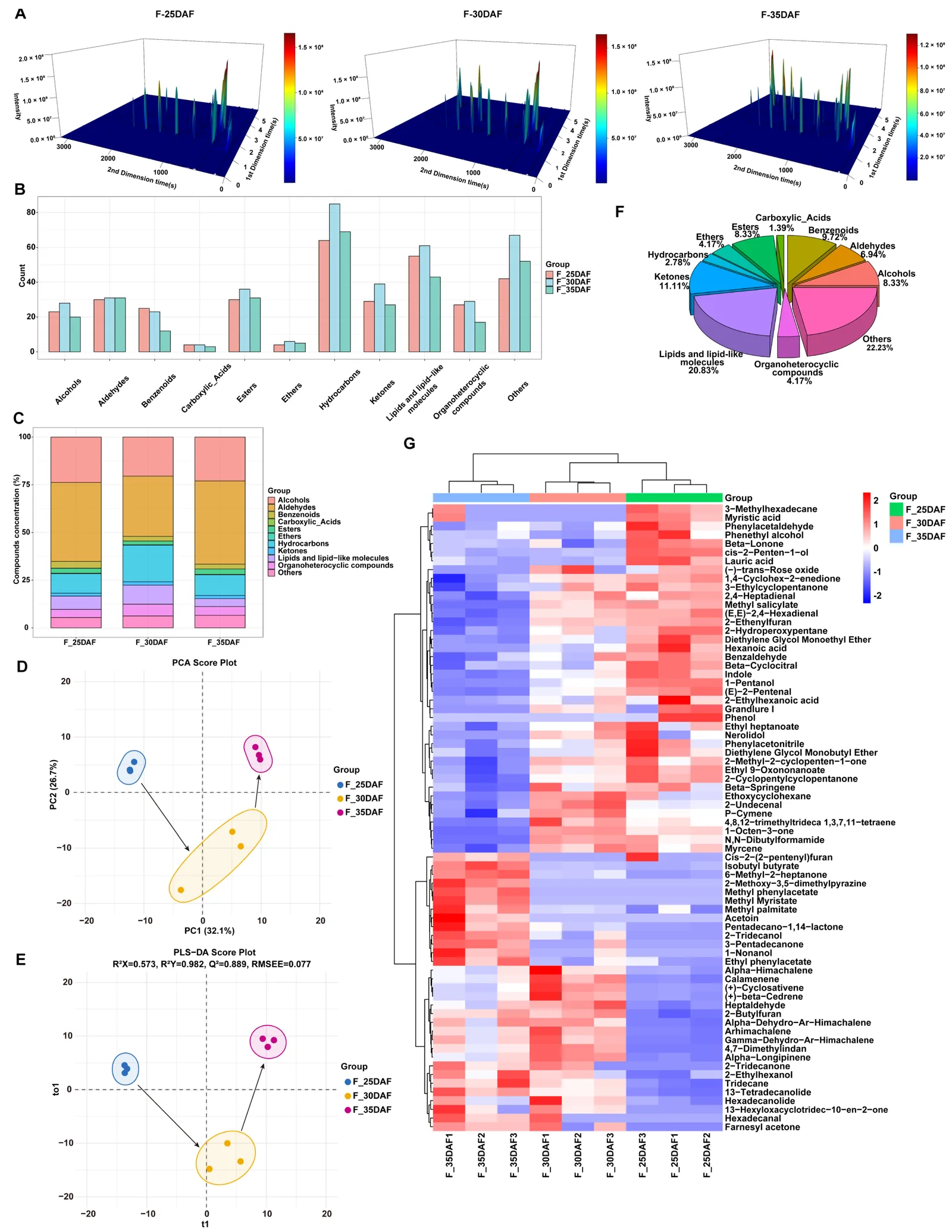
VOC analysis of fruity-aroma pitaya at 25, 30 and 35 DAF. (**A**) Three-dimensional chromatograms. (**B**) Number of VOCs in each class. (**C**) Relative abundance of each VOC class. (**D**) PCA score plot. (**E**) PLS-DA score plot. (**F**) The pie chart of the proportion of key differential VOC classes. (**G**) The cluster heatmap of key differential VOCs.

VOCs in the fruity-aroma pitaya at 25, 30 and 35 DAF were also analyzed using GC × GC-TOF-MS, and the results are presented as three-dimensional chromatograms (Figure 3A). Figure 3B,C show the number and relative abundances of VOCs in the fruity-aroma pitaya at different ripening stages, respectively. At 25 DAF, 333 VOCs were identified, comprising 23 alcohols, 30 aldehydes, 25 benzeneoids, 4 carboxylic acids, 30 esters, 4 ethers, 64 hydrocarbons, 29 ketones, 55 lipids and lipid-like molecules, 27 organoheterocyclic compounds, and 42 other compounds. At 30 DAF, 409 VOCs were detected, including 28 alcohols, 31 aldehydes, 23 benzeneoids, 4 carboxylic acids, 36 esters, 6 ethers, 85 hydrocarbons, 39 ketones, 61 lipids and lipid-like molecules, 29 organoheterocyclic compounds, and 67 other compounds. At 35 DAF, 310 VOCs were identified, consisting of 20 alcohols, 31 aldehydes, 12 benzeneoids, 3 carboxylic acids, 31 esters, 5 ethers, 69 hydrocarbons, 27 ketones, 43 lipids and lipid-like molecules, 17 organoheterocyclic compounds, and 52 other compounds (Figure 3B).

The relative abundance of VOCs in the fruity-aroma pitaya varied across developmental stages. Aldehydes exhibited the highest relative content throughout the ripening process, initially decreasing before increasing again. Alcohols were the second most abundant, also showing a decrease followed by an increase. Hydrocarbons, lipids and lipid-like molecules, and organoheterocyclic compounds initially increased, then decreased. Benzenoids decreased initially before stabilizing. Esters and ketones were at low levels and did not change significantly. Carboxylic acids and ethers stayed at consistently very low levels throughout the ripening process. These findings suggest that the volatile profile of the fruity-aroma pitaya underwent significant changes over time during ripening (Figure 3C).

#### 3.2.2. Analysis of Key VOCs in Fruity-Aroma Pitaya

The PCA score plot revealed significant separation of VOCs at different ripening stages (Figure 3D). The first two principal components (PC1 and PC2) together explained 58.8% of the total variation, with PC1 contributing 32.1% and PC2 contributing 26.7%. This result suggested that the volatile profile of the fruity-aroma pitaya at 25, 30 and 35 DAF differs significantly. Then, the PLS-DA score plot further demonstrated clear differentiation between samples at different ripening stages (Figure 3E), with the following parameters: R^2^X = 0.573, R^2^Y = 0.982, Q^2^ = 0.889, and RMSEE = 0.077. In summary, both PCA and PLS-DA analyses consistently indicated that VOC differences became more distinct as the fruity-aroma pitaya ripened.

Using VIP > 1 and *p* < 0.05 as screening criteria, a total of 72 differentially abundant VOCs were identified at 25, 30 and 35 DAF, including 6 alcohols, 5 aldehydes, 7 benzeneoids, 1 carboxylic acid, 6 esters, 3 ethers, 2 hydrocarbons, 8 ketones, 15 lipids and lipid-like molecules, 3 organoheterocyclic compounds, and 16 other compounds. Lipids and lipid-like molecules (20.83%), ketones (11.11%) and benzeneoids (9.72%) were the major differentially expressed components, followed by esters (8.33%), alcohols (8.33%), aldehydes (6.94%), ethers (4.17%) and organoheterocyclic compounds (4.17%) (Figure 3F).

The cluster heatmap analysis revealed significant changes in relative VOC abundances across the ripening stages (Figure 3G). As ripening progressed, the levels of phenylacetaldehyde, hexanoic acid, 1-pentanol, cis-2-penten-1-ol, (E, E)-2,4-hexadienal, (E)-2-pentenal, benzaldehyde and phenylacetonitrile gradually decreased. In contrast, 13-tetradecanolide, 2-ethylhexanol, ethyl phenylacetate, tridecane and pentadecano-1,14-lactone gradually increased. In addition, alpha-longipinene, heptaldehyde, (+)-cyclosativene, ethoxycyclohexane, (+)-beta-cedrene, p-cymene, and 1-octen-3-one initially increased and then decreased. For example, the FDR-adjusted *p*-values for 1-pentanol, (E)-2-pentenal, 13-tetradecanolide, and ethyl phenylacetate were 3.43 × 10^−5^, 2.32 × 10^−4^, 0.0017, and 0.0201, respectively. These results suggest that the VOC composition of the fruity-aroma pitaya undergoes significant alterations during ripening.

#### 3.2.3. ROAV Analysis in Fruity-Aroma Pitaya

Table 2 lists the potential aroma-active compounds with an ROAV ≥ 0.1 in the fruity-aroma pitaya at different ripening stages. At 25, 30 and 35 DAF, 11, 9 and 9 compounds exhibited ROAVs above 0.1, respectively. Among these, 2-nonenal (ROAV = 100), with its fatty, cucumber-like aroma, was the most prominent contributor throughout ripening. The ROAV of heptaldehyde, which has citrus, fatty and rancid notes, decreased significantly over time. Additionally, the ROAVs of 2-pentylfuran and (E)-2-octenal were markedly reduced at 35 DAF, which may account for the diminished grassy aroma. (E)-2-octenal may be closely associated with the description of “green and grassy” [35]. Meanwhile, heptaldehyde, 2-pentylfuran and (E)-2-octenal were also the aroma-active compounds in typical oriental melon varieties [36]. Similarly, the ROAVs of 2-ethylhexanol, 2-dodecenal, (2E,6Z)-nona-2,6-dienal, and 1-hexanol were all present at relatively low levels at 35 DAF, which may also have contributed to the reduction in the grassy aroma. A study found that among the VOCs of different plum varieties, 1-hexanol had the highest content [37]. In addition, “sour/rancid” is linked to isovaleric acid [38]. We found that isovaleric acid has a rancid cheese, sweaty, and putrid odor, and 1-octen-3-one has a mushroom-like odor. Both showed a significant decrease over time, thereby reducing their impact. Moreover, phenylacetaldehyde was present in higher abundances in unripe pear fruits and could produce a “green” odor [39]. In this study, phenylacetaldehyde initially decreased and then stabilized, developing a floral, honey-like aroma that might have enhanced the fruity aroma. Therefore, these VOCs might help reduce the grassy aroma while enhancing the fruity aroma.

### 3.3. Comparative Analysis Between Herbaceous and Fruity Cultivars

#### 3.3.1. Early Development (25 DAF)

**Figure 4:**
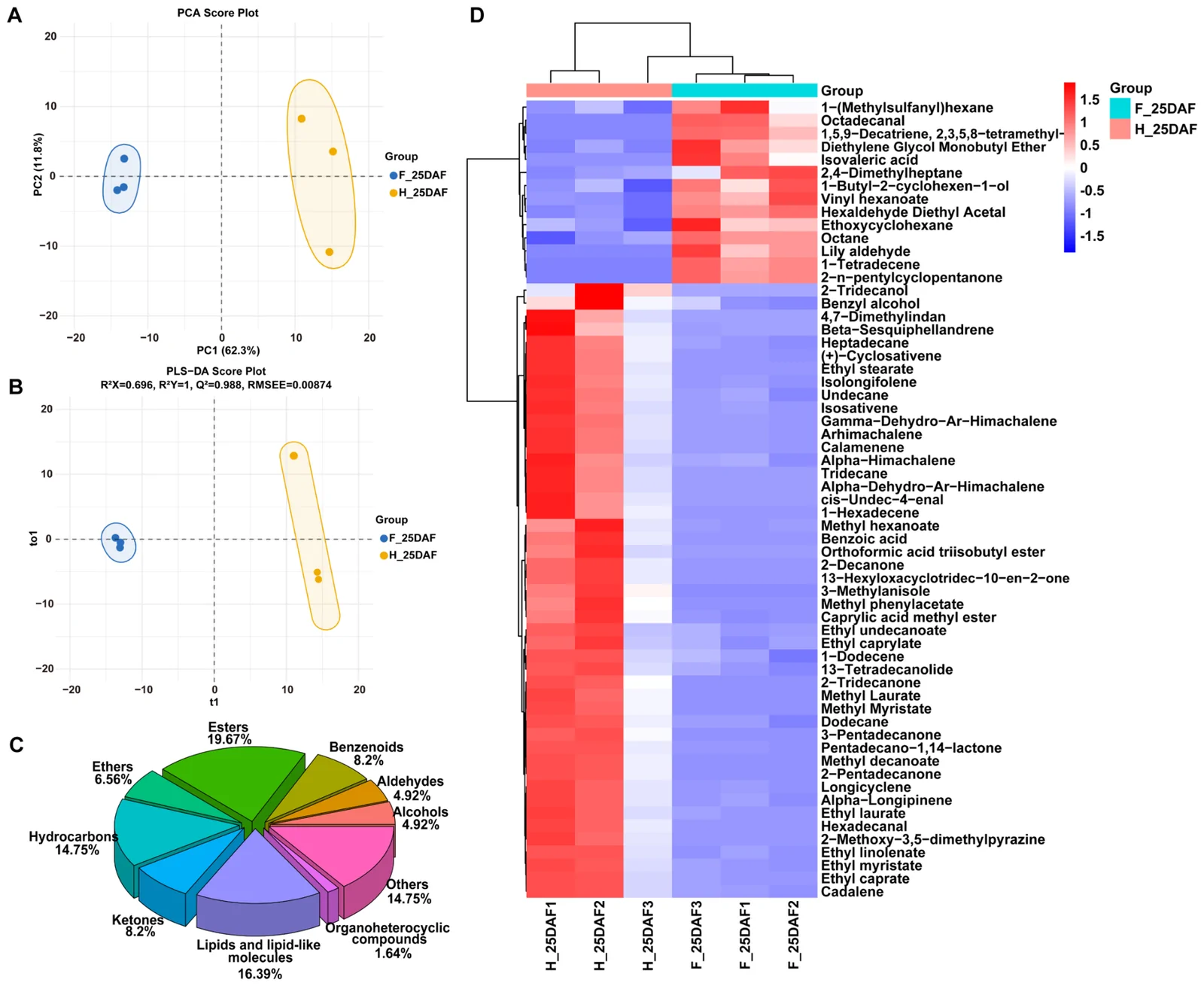
VOC analysis of herbaceous-/fruity-aroma pitaya at 25 DAF. (**A**) PCA score plot. (**B**) PLS-DA score plot. (**C**) The pie chart of the proportion of key differential VOC classes. (**D**) The cluster heatmap of key differential VOCs.

To investigate the causes of aroma differences, we compared the VOCs and ROAVs of two cultivars of pitaya with herbaceous and fruity aromas at the same ripening stage. At 25 DAF, the PCA score plot revealed significant separation in their VOCs (Figure 4A). The first two principal components (PC1 and PC2) collectively explained 74.1% of the total variance, with PC1 accounting for 62.3% and PC2 for 11.8%. The results indicate that at 25 DAF, the volatile profiles of the herbaceous and fruity varieties differed markedly. PLS-DA was further applied to assess VOC differences between the varieties. The PLS-DA score plot also demonstrated clear differentiation at 25 DAF (Figure 4B), with the following parameters: R^2^X = 0.696, R^2^Y = 1, Q^2^ = 0.988, and RMSEE = 0.00874. In summary, PCA and PLS-DA analyses consistently showed that the aroma characteristics of the herbaceous-/fruity-aroma pitaya fruits were significantly different at 25 DAF.

Based on the screening criteria of VIP > 1 and *p* < 0.05, a total of 61 significantly differentially abundant VOCs were identified at 25 DAF. These included 3 alcohols, 3 aldehydes, 5 benzeneoids, 12 esters, 4 ethers, 9 hydrocarbons, 5 ketones, 10 lipids and lipid-like molecules, 1 organoheterocyclic compound, and 9 other compounds. Esters (19.67%), lipids and lipid-like molecules (16.39%), and hydrocarbons (14.75%) were the major differentially expressed components, followed by ketones (8.2%), benzenoids (8.2%), ethers (6.56%), aldehydes (4.92%) and alcohols (4.98%) (Figure 4C).

The cluster heatmap shows that the relative VOC abundances showed significant differences at 25 DAF (Figure 4D). Notably, the levels of tridecane, ethyl caprylate, alpha-longipinene, dodecane, 13-tetradecanolide, ethyl laurate, undecane, longicyclene, (+)-cyclosativene, ethyl caprate, 2-tridecanol, and pentadecano-1,14-lactone were significantly higher in the herbaceous-aroma pitaya than in the fruity-aroma pitaya. Conversely, the levels of isovaleric acid, 2-n-pentylcyclopentanone, vinyl hexanoate, diethylene glycol monobutyl ether, 2,4-dimethylheptane, and octane were significantly higher in the fruity-aroma pitaya than in the herbaceous-aroma pitaya. For example, the FDR-adjusted *p*-values for 2-n-pentylcyclopentanone and vinyl hexanoate were 0.00358 and 0.02428, respectively. These results suggest that the aroma characteristics of pitaya with an herbaceous aroma and with a fruity aroma differ significantly at 25 DAF.

Table 3 lists the potential aroma-active compounds with an ROAV ≥ 0.1 at 25 DAF in both the herbaceous-aroma and the fruity-aroma pitaya varieties. In the herbaceous-/fruity-aroma pitaya, 6 and 11 compounds had ROAVs above this threshold, respectively. Among these, 2-nonenal was the most prominent contributor in both pitaya aroma varieties. Heptaldehyde, 2-pentylfuran, and (E)-2-octenal followed, with significantly higher ROAVs in the fruity-aroma pitaya than in the herbaceous-aroma pitaya. Moreover, 1-octen-3-one, isovaleric acid, 2-dodecenal, phenylacetaldehyde, 1-hexanol, (2E,6Z)-nona-2,6-dienal, and 2-ethylhexanol also contributed to the fruity aroma. In contrast, 2-dodecenal and (2E,6Z)-nona-2,6-dienal contributed to the herbaceous aroma. Since the pitaya was inedible at 25 DAF, we further compared the ROAV contributors at subsequent ripening stages.

#### 3.3.2. Transition Phase (30 DAF)

**Figure 5:**
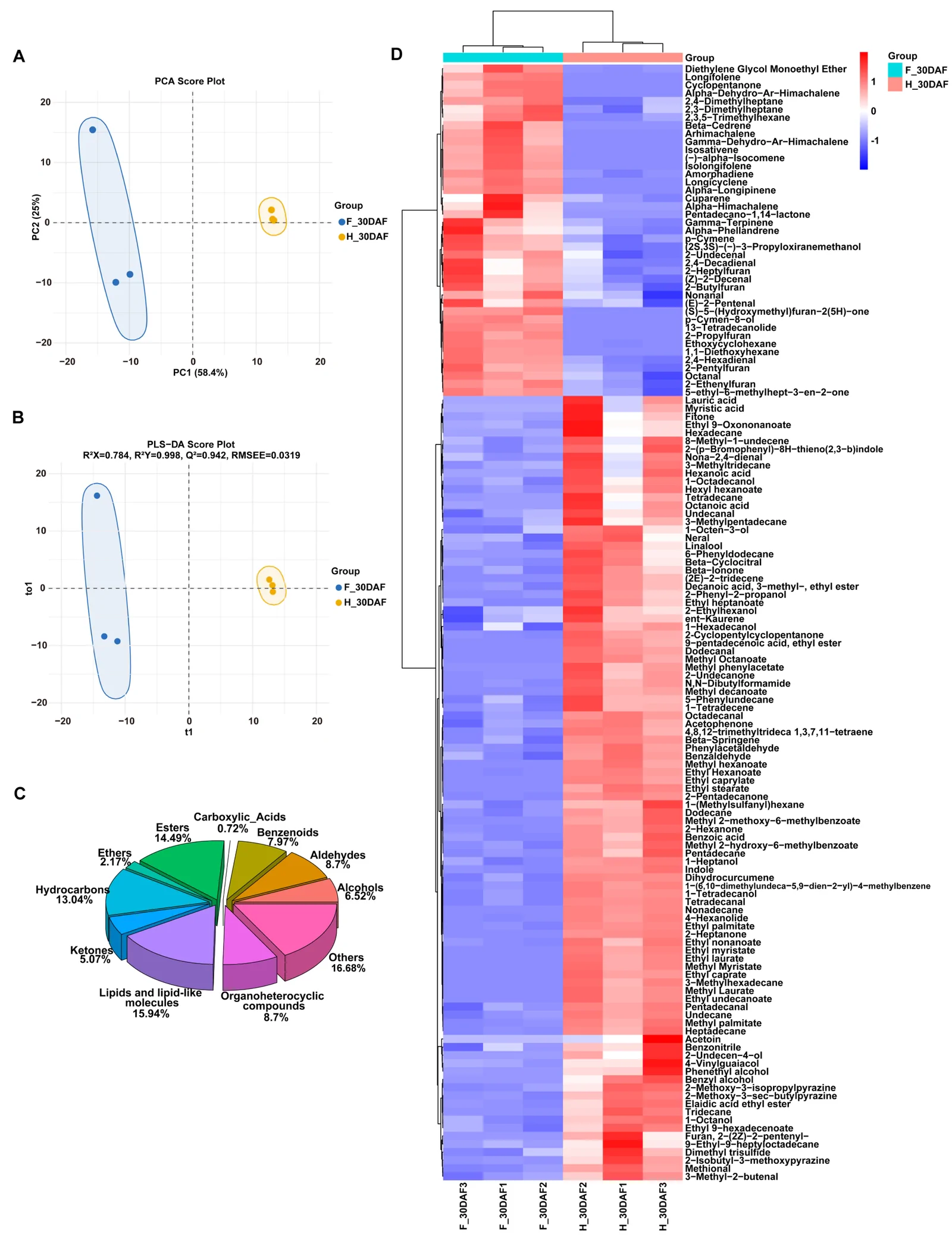
VOC analysis of herbaceous-/fruity-aroma pitaya at 30 DAF. (**A**) PCA score plot. (**B**) PLS-DA score plot. (**C**) The pie chart of the proportion of key differential VOC classes. (**D**) The cluster heatmap of key differential VOCs.

We further compared the VOCs and ROAVs of the herbaceous-/fruity-aroma pitaya at 30 DAF. The PCA score plot showed clear separation of the VOCs between the two varieties (Figure 5A). The first two principal components (PC1 and PC2) explained 83.4% of the total variance, with PC1 accounting for 58.4% and PC2 for 25%. These results indicate that the volatile profiles of the herbaceous/fruity-aroma pitaya differed significantly at 30 DAF. The PLS-DA score plot further confirmed clear differentiation in the VOCs at 30 DAF (Figure 5B), with the following parameters: R^2^X = 0.784, R^2^Y = 0.998, Q^2^ = 0.942, and RMSEE = 0.0319. In short, both PCA and PLS-DA analyses consistently showed significant differences in the volatile profiles of the herbaceous-/fruity-aroma pitaya at 30 DAF.

According to the screening criteria of VIP > 1 and *p* < 0.05, a total of 138 significantly differentially abundant VOCs were identified at 30 DAF, including 9 alcohols, 12 aldehydes, 11 benzeneoids, 1 carboxylic acid, 20 esters, 3 ethers, 18 hydrocarbons, 7 ketones, 22 lipids and lipid-like molecules, 12 organoheterocyclic compounds, and 23 other compounds. Lipids and lipid-like molecules (15.94%), esters (14.49%), and hydrocarbons (13.04%) were the major differentially expressed components, followed by aldehydes (8.7%), organoheterocyclic compounds (8.7%), benzenoids (7.97%), alcohols (6.52%), and ketones (5.07%) (Figure 5C).

As shown in Figure 5D, there were significant differences in the relative VOC abundances between the two varieties at 30 DAF. The abundances of octanoic acid, hexanoic acid, 2-heptanone, phenylacetaldehyde, 1-tetradecanol, ethyl caprylate, tridecane, ethyl hexanoate, acetoin, 2-ethylhexanol, tetradecanal, ethyl myristate, ethyl laurate, ethyl caprate, dodecane, benzaldehyde, undecane, 1-octanol, and 2-undecanone were significantly higher in the herbaceous-aroma pitaya than in the fruity-aroma pitaya. Conversely, the abundances of 2-pentylfuran, 2,4-dimethylheptane, longifolene, alpha-longipinene, 2,4-hexadienal, 13-tetradecanolide, longicyclene, octanal, and 2,3,5-trimethylhexane were significantly higher in pitaya with a fruity aroma than in pitaya with an herbaceous aroma. For example, the FDR-adjusted *p*-values for 2-heptanone, ethyl caprylate, 2-pentylfuran, and 13-tetradecanolide were 8.93 × 10^−5^, 2.89 × 10^−4^, 0.00486, and 8.93 × 10^−5^, respectively. These results indicate significant differences in the volatile profiles of pitaya with herbaceous and fruity aromas at 30 DAF.

Potential aroma-active compounds with ROAVs ≥ 0.1 at 30 DAF in the herbaceous-/fruity-aroma pitaya varieties are listed in Table 4. In the herbaceous-/fruity-aroma pitaya, 11 and 9 compounds showed ROAVs above 0.1, respectively. Among them, 2-nonenal was the most prominent contributor in both varieties. Heptaldehyde, characterized by citrus, fatty, and rancid notes, also played a major role. Compounds such as 2-pentylfuran and (E)-2-octenal, both associated with green notes, exhibited higher ROAVs and likely contributed to the herbaceous aroma. Similarly, 2-undecanone, 2-ethylhexanol, (2E,6Z)-nona-2,6-dienal, and 1-hexanol, all with green characteristics, are more abundant in the herbaceous-aroma pitaya than in the fruity-aroma pitaya. In contrast, isovaleric acid and 2-dodecenal were higher in the fruity-aroma pitaya, while 2-heptanone and phenylacetaldehyde were higher in the herbaceous-aroma pitaya. These findings highlight distinct differences in the volatile profiles of herbaceous and fruity pitaya at 30 DAF.

#### 3.3.3. Fully Ripe Stage (35 DAF)

**Figure 6:**
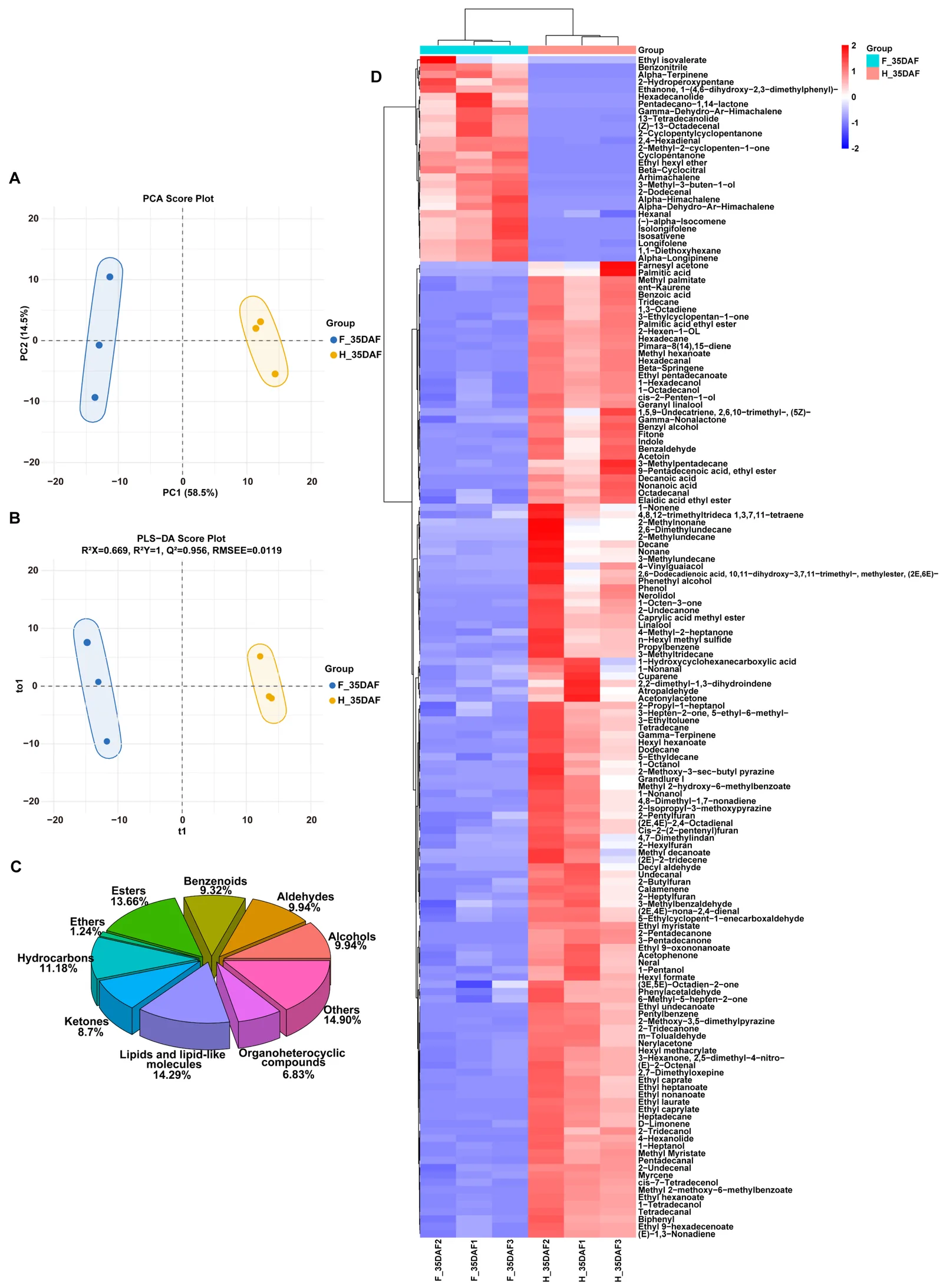
VOC analysis of herbaceous-/fruity-aroma pitaya at 35 DAF. (**A**) PCA score plot. (**B**) PLS-DA score plot. (**C**) The pie chart of the proportion of key differential VOC classes. (**D**) The cluster heatmap of key differential VOCs.

We also compared the VOCs and ROAVs of the herbaceous-/fruity-aroma pitaya at 35 DAF. As shown in the PCA score plot (Figure 6A), the two cultivars exhibited distinct separation in their VOC profiles. The first two principal components (PC1 and PC2) explained 73% of the total variation, with PC1 accounting for 58.5% and PC2 for 14.5%. Similarly, the PLS-DA score plot also demonstrated distinct separation between the two varieties (Figure 6B), with R^2^X = 0.669, R^2^Y = 1, Q^2^ = 0.956, and RMSEE = 0.0119. These results showed significant differences in the volatile profiles of the herbaceous-/fruity-aroma pitaya at 35 DAF.

Based on the screening criteria of VIP > 1 and *p* < 0.05, 161 significantly differentially abundant VOCs were identified at 35 DAF. These included 16 alcohols, 16 aldehydes, 15 benzeneoids, 22 esters, 2 ethers, 18 hydrocarbons, 14 ketones, 23 lipids and lipid-like molecules, 11 organoheterocyclic compounds, and 24 other compounds. Lipids and lipid-like molecules (14.29%), esters (13.66%), and hydrocarbons (11.18%) represented the major differentially expressed classes, followed by alcohols (9.94%), aldehydes (9.94%), benzeneoids (9.32%), ketones (8.70%), and organoheterocyclic compounds (6.83%) (Figure 6C).

As shown in Figure 6D, at 35 DAF, the two pitaya varieties exhibited distinct differences in relative VOC abundances. The herbaceous-aroma pitaya contained significantly higher levels of acetoin, tridecane, 1-octanol, ethyl caprylate, dodecane, phenylacetaldehyde, 1-tetradecanol, 2-hexen-1-ol, tetradecanal, 2-pentylfuran, ethyl hexanoate, ethyl myristate, ethyl laurate, decane, 5-ethylcyclopent-1-enecarboxaldehyde, ethyl caprate, tetradecane, 1-hexadecanol, methyl 2-hydroxy-6-methylbenzoate, 2-tridecanol, benzaldehyde, 1-heptanol, (E)-2-octenal, 2-undecanone, and 1-octen-3-one compared with the fruity-aroma pitaya. Conversely, hexanal, 13-tetradecanolide, alpha-longipinene, longifolene, 3-methyl-3-buten-1-ol, and 2-dodecenal were present at significantly higher abundances in the fruity-aroma pitaya. For example, the FDR-adjusted *p*-values for (E)-2-octenal, 2-undecanone, hexanal, and 13-tetradecanolide were 0.00367, 0.00943, 0.00724, and 0.00371, respectively. These results demonstrate that the two cultivars exhibited markedly different VOC profiles at 35 DAF, highlighting potential differences in aroma-related compounds.

Table 5 presents the potential aroma-active compounds (ROAV ≥ 0.1) at 35 DAF in the herbaceous-/fruity-aroma pitaya. In total, 12 compounds were identified in the herbaceous-aroma pitaya and 9 in the fruity-aroma pitaya. 2-Nonenal was the most dominant contributor in both varieties. Heptaldehyde, characterized by citrus, fatty, and rancid notes, also showed elevated levels in both varieties, highlighting its importance in overall aroma formation. Notably, 2-pentylfuran and (E)-2-octenal displayed significantly higher ROAVs in the herbaceous-aroma pitaya than in the fruity-aroma pitaya. Both compounds impart a green odor and might serve as the primary contributors to the herbaceous aroma. In addition, 2-ethylhexanol, (2E,6Z)-nona-2,6-dienal, 1-hexanol, and 2-undecanone exhibited higher ROAVs in the herbaceous-aroma pitaya, suggesting that these greenish-scented compounds act as secondary contributors. Some studies revealed that (2E,6Z)-nona-2,6-dienal is the main aroma component of oriental melons (ZhongHuaCaiGua) and Jiashi muskmelon juice [40,41]. Other compounds, including 2-heptanone, 1-octen-3-one, phenylacetaldehyde, and 1-octanol, also showed elevated ROAVs in the herbaceous-aroma pitaya, likely enhancing aroma complexity. 1-Octanol was also found in yellow passion fruit essence [42]. In contrast, 2-dodecenal might contribute more strongly to the fruity aroma. Meanwhile, 2-dodecenal is one of the main aroma components of passion fruit (*Passiflora alata* Ait.) [43]. Overall, these findings indicate pronounced differences in volatile profiles between the herbaceous- and fruity-aroma pitaya fruit at 35 DAF. For both cultivars, full ripeness at 35 DAF indicated an appropriate harvest stage for fresh consumption. The cultivar- and maturity-dependent VOC profiles may also provide useful guidance for aroma-oriented raw material selection in food processing.

## 4. Discussion

This study successfully screened for the differentially expressed substances contributing to the different aromas of these two pitaya fruit varieties using GC × GC-TOF-MS. These differences do not originate from substances specific to a particular variety, but are driven by highly differentiated development-dependent variation within a shared biosynthetic network.

It should be noted that the odor thresholds used for ROAV calculations were obtained from published literature and may not fully reflect the characteristics of the pitaya pulp matrix. Matrix components such as sugars, pectin, and organic acids may influence VOC partitioning and release, thereby affecting their perceived aroma contributions. Therefore, the calculated ROAVs should be regarded as estimates of potential aroma relevance rather than definitive measures of sensory impact. In this study, ROAV analysis identified several potentially aroma-relevant compounds, including 2-pentylfuran, (E)-2-octenal, and 2-undecanone. However, their individual contributions to pitaya aroma remain unverified. Future studies combining GC-olfactometry, aroma recombination, and omission experiments are necessary to establish their actual sensory significance.

The differences in herbaceous and fruity aromas might be associated with the lipoxygenase (LOX) pathway. The abundant accumulation of (E)-2-octenal and 2-pentylfuran in the herbaceous pitaya fruit constitutes its signature metabolic characteristic. The biosynthesis of these compounds is closely related to the LOX pathway [44,45,46]. During fruit ripening, membrane lipid metabolism releases polyunsaturated fatty acids (such as linoleic acid and linolenic acid), which are then stereoselectively oxidized by LOX. Subsequently, hydroperoxide lyase (HPL) cleaves these fatty acids, generating highly reactive, pungent aldehydes such as (E)-2-octenal [44,45,47]. These unsaturated aldehydes then undergo intramolecular cyclization and further oxidation to generate stable furan derivatives, most notably 2-pentylfuran [46,48]. At 25 to 35 days post-flowering, the ROAV values of these compounds continued to rise, suggesting the potential involvement of the LOX pathway during the late ripening stage. However, in many aromatic fruits, the strong upregulation of alcohol dehydrogenases (ADHs) and alcohol acyltransferases (AATs) rapidly converts pungent aldehydes into sweeter alcohols and esters [44,45,49]. Our results suggest that the fruity “Yuehong No. 6” might effectively utilize this ADH/AAT pathway to neutralize its herbaceous aroma, while the herbaceous “Vietnamese White Heart” encounters a metabolic bottleneck, leading to the accumulation of LOX-derived green volatiles.

The difference between herbaceous and fruity aromas might also be due to the presence of specific substances in the β-oxidation pathway. This study found that 2-undecanone was also a marker of herbaceous aromas. It is generated from fatty acid β-oxidation and typically imparts complex, creamy and fruity aromas to microbially fermented foods [17,47,50]. However, its high-level, stable expression in fresh edible pulp is evolutionarily uncommon and highly specific. In the herbaceous pitaya fruit, 2-undecanone accumulated stably at full ripeness, with an ROAV value of 1.94, while in fruity pitaya fruit, it was almost completely suppressed (ROAV value of 0.04). It gives pitaya fruit a fresh and green aroma, and works perfectly with LOX derivatives, greatly enriching the complexity of the herbaceous aroma. Therefore, the herbaceous aroma may be associated with differences in LOX-related lipid oxidation and β-oxidation pathways, while the fruity aroma of “Yuehong No. 6” not only suppresses green volatiles but also shows active accumulation of specific sweet, floral and fruity components. The retention and accumulation of several esters support its fruity characteristics. For example, 2-dodecenal (a compound that imparts a sweet aroma) was present in significantly higher amounts in the “Yuehong No. 6” variety. Therefore, the synergistic effect of these substances creates the unique aroma characteristics of the “Yuehong No. 6” pitaya fruit.

Although the observed VOC profiles suggest the potential involvement of the LOX, β-oxidation, and ADH/AAT pathways in cultivar-specific aroma formation, these proposed mechanisms remain to be experimentally validated. Future studies integrating transcriptomic analyses (e.g., RNA-seq) and enzyme activity assays targeting LOX, ADH, and AAT will be necessary to further elucidate the underlying metabolic regulation.

Because the LOX and β-oxidation pathways are highly sensitive to environmental stimuli, targeted postharvest interventions can be implemented [51,52,53,54,55]. For example, supplemental LED lighting with a specific spectrum can significantly modulate the secondary metabolism of pitaya fruit and regulate its ripening cascade [56,57]. This controlled environmental strategy can be optimized to upregulate ADH/AAT activity, thereby reducing herbaceous flavor and increasing sweetness. Furthermore, this study lays the foundation for molecular breeding. By mapping specific volatile biomarkers (e.g., (E)-2-octenal, 2-pentylfuran, and 2-undecanone) to LOX gene loci, future breeding programs (including CRISPR/Cas9 gene editing) can precisely knock out or overexpress these loci. This allows for the customization of more delicious and popular pitaya fruit according to consumer preferences without compromising nutritional value.

## 5. Conclusions

This study used GC × GC-TOF-MS to characterize cultivar- and developmental stage-dependent variation in VOC profiles between herbaceous- and fruity-aroma pitaya varieties. The results revealed significant differences in both VOCs and ROAVs between the herbaceous-/fruity-aroma pitaya across different maturity stages. Notably, distinct variations were also observed between the two varieties at the same developmental stage. The aroma of the herbaceous pitaya fruit was primarily associated with the excessive accumulation of 2-pentylfuran, (E)-2-octenal and 2-undecanone. In contrast, fruity-aroma pitaya not only reduced these strongly green aroma compounds but also specifically accumulated sweet components (such as 2-dodecenal). For both “Vietnamese White Heart” and “Yuehong No. 6”, full ripeness at 35 DAF serves as a practical harvest indicator for fresh consumption. For industrial processing, the observed cultivar- and maturity-dependent VOC differences may guide raw material selection based on specific aroma requirements, while the optimal harvest maturity should be determined according to the intended processing application. This research lays a solid foundation for targeted genomics-assisted precision breeding, postharvest intervention, flavor-oriented metabolic engineering, and the development and processing of pitaya fruit-derived functional foods in the global pitaya fruit market.

## Figures and Tables

**Table 1:** ROAVs of potentially aroma-relevant VOCs in herbaceous-aroma pitaya at 25, 30 and 35 DAF.

Name	Odor Character	H_25DAF_ROAV	H_30DAF_ROAV	H_35DAF_ROAV
2-Nonenal	Fatty, Cucumber	100.00	100.00	100.00
2-Pentylfuran	Green Beans, Vegetable	5.31	10.79	19.83
(E)-2-Octenal	Nuts, Green, Fatty	2.35	4.82	10.83
Heptaldehyde	Citrus, Fatty, Rancid	6.27	13.75	10.01
2-Undecanone	Orange, Fresh, Green	0.09	0.51	1.94
1-Octen-3-one	Mushroom-Like	0.09	0.32	0.96
2-Heptanone	Sweet, Mushroom	0.03	0.57	0.51
1-Octanol	Penetrating	0.01	0.04	0.39
1-Hexanol	Green Grass, Plastic	0.04	0.15	0.29
2-Ethylhexanol	Rose, Green	0.08	0.25	0.27
(2E,6Z)-nona-2,6-dienal	Cucumber, Green	0.35	0.25	0.22
Phenylacetaldehyde	Floral, Honey	0.06	0.31	0.21
2-Dodecenal	Green, Fatty, Sweet	0.15	<0.01	<0.01

Abbreviation: H, the herbaceous-aroma cultivar “Vietnamese White Heart”; DAF, days after flowering; ROAV, relative odor activity value.

**Table 2:** ROAVs of potentially aroma-relevant VOCs in fruity-aroma pitaya at 25, 30 and 35 DAF.

Name	Odor Character	F_25DAF_ROAV	F_30DAF_ROAV	F_35DAF_ROAV
2-Nonenal	Fatty, Cucumber	100.00	100.00	100.00
Heptaldehyde	Citrus, Fatty, Rancid	19.98	12.87	10.55
2-Pentylfuran	Green Beans, Vegetable	18.67	18.16	8.97
(E)-2-Octenal	Nuts, Green, Fatty	8.29	6.56	3.29
1-Octen-3-one	Mushroom-Like	1.27	0.59	0.19
2-Ethylhexanol	Rose, Green	0.11	0.11	0.16
2-Dodecenal	Green, Fatty, Sweet	0.39	0.23	0.16
(2E,6Z)-nona-2,6-dienal	Cucumber, Green	0.21	0.19	0.15
1-Hexanol	Green Grass, Plastic	0.23	0.10	0.13
Phenylacetaldehyde	Floral, Honey	0.33	0.09	0.09
Isovaleric acid	Rancid Cheese, Sweaty, Putrid	0.51	0.20	0.04

Abbreviation: F, the fruity-aroma cultivar “Yuehong No. 6”; DAF, days after flowering; ROAV, relative odor activity value.

**Table 3:** ROAVs of potentially aroma-relevant VOCs in herbaceous-/fruity-aroma pitaya at 25 DAF.

Name	Odor Character	F_25DAF_ROAV	H_25DAF_ROAV
2-Nonenal	Fatty, Cucumber	100.00	100.00
Heptaldehyde	Citrus, Fatty, Rancid	19.98	6.27
2-Pentylfuran	Green Beans, Vegetable	18.67	5.31
(E)-2-Octenal	Nuts, Green, Fatty	8.29	2.35
1-Octen-3-one	Mushroom-Like	1.27	0.09
Isovaleric acid	Rancid Cheese, Sweaty, Putrid	0.51	<0.01
2-Dodecenal	Green, Fatty, Sweet	0.39	0.15
Phenylacetaldehyde	Floral, Honey	0.33	0.06
1-Hexanol	Green Grass, Plastic	0.23	0.04
(2E,6Z)-nona-2,6-dienal	Cucumber, Green	0.21	0.35
2-Ethylhexanol	Rose, Green	0.11	0.08

Abbreviation: F, the fruity-aroma cultivar “Yuehong No. 6”; H, the herbaceous-aroma cultivar “Vietnamese White Heart”; DAF, days after flowering; ROAV, relative odor activity value.

**Table 4:** ROAVs of potentially aroma-relevant VOCs in herbaceous-/fruity-aroma pitaya at 30 DAF.

Name	Odor Character	F_30DAF_ROAV	H_30DAF_ROAV
2-Nonenal	Fatty, Cucumber	100.00	100.00
2-Pentylfuran	Green Beans, Vegetable	18.16	10.79
Heptaldehyde	Citrus, Fatty, Rancid	12.87	13.75
(E)-2-Octenal	Nuts, Green, Fatty	6.56	4.82
1-Octen-3-one	Mushroom-Like	0.59	0.32
2-Dodecenal	Green, Fatty, Sweet	0.23	<0.01
Isovaleric acid	Rancid Cheese, Sweaty, Putrid	0.20	0.06
(2E,6Z)-nona-2,6-dienal	Cucumber, Green	0.19	0.25
2-Ethylhexanol	Rose, Green	0.11	0.25
1-Hexanol	Green Grass, Plastic	0.10	0.15
Phenylacetaldehyde	Floral, Honey	0.09	0.31
2-Undecanone	Orange, Fresh, Green	0.04	0.51
2-Heptanone	Sweet, Mushroom	0.01	0.57

Abbreviation: F, the fruity-aroma cultivar “Yuehong No. 6”; H, the herbaceous-aroma cultivar “Vietnamese White Heart”; DAF, days after flowering; ROAV, relative odor activity value.

**Table 5:** ROAVs of potentially aroma-relevant VOCs in herbaceous-/fruity-aroma pitaya at 35 DAF.

Name	Odor Character	F_35DAF_ROAV	H_35DAF_ROAV
2-Nonenal	Fatty, Cucumber	100.00	100.00
2-Pentylfuran	Green Beans, Vegetable	8.97	19.83
(E)-2-Octenal	Nuts, Green, Fatty	3.29	10.83
Heptaldehyde	Citrus, Fatty, Rancid	10.55	10.01
2-Undecanone	Orange, Fresh, Green	0.04	1.94
1-Octen-3-one	Mushroom-Like	0.19	0.96
2-Heptanone	Sweet, Mushroom	0.01	0.51
1-Octanol	Penetrating	0.01	0.39
1-Hexanol	Green Grass, Plastic	0.13	0.29
2-Ethylhexanol	Rose, Green	0.16	0.27
(2E,6Z)-nona-2,6-dienal	Cucumber, Green	0.15	0.22
Phenylacetaldehyde	Floral, Honey	0.09	0.21
2-Dodecenal	Green, Fatty, Sweet	0.16	<0.01

Abbreviation: F, the fruity-aroma cultivar “Yuehong No. 6”; H, the herbaceous-aroma cultivar “Vietnamese White Heart”; DAF, days after flowering; ROAV, relative odor activity value.

## Data Availability

The original contributions presented in this study are included in the article. Further inquiries can be directed to the corresponding authors.

## Abbreviations

The following abbreviations are used in this manuscript:

DAF	Days after flowering
GC × GC-TOF-MS	Gas chromatography with time-of-flight mass spectrometric detection
PCA	Principal component analysis
PLS-DA	Partial least squares discriminant analysis
ROAV	Relative odor activity value
VOCs	Volatile organic compounds
